# T4 reduces cisplatin resistance by inhibiting AEG-1 gene expression in lung cancer cells

**DOI:** 10.1038/s41598-022-15643-3

**Published:** 2022-07-06

**Authors:** Tian-jiao Song, Xiao-hong Lin, Ping-ting Huang, Yu-qing Chen, Li-min Chen

**Affiliations:** 1 Department of Respiratory Medicine, Affiliated Union Hospital of Fujian Medical University, No.29 Xinquan Road, Fuzhou, 350001 Fujian People’s Republic of China; 2 Department of Emergency, Fujian Provincial Hospital, 134 East Street, Fuzhou, 350001 Fujian People’s Republic of China; 3 Shengli Clinical Medical College of Fujian Medical University, 134 East Street, Fuzhou, 350001 Fujian People’s Republic of China; 4 Fujian Provincial Key Laboratory of Emergency Medicine, 134 East Street, Fuzhou, 350001 Fujian People’s Republic of China; 5 Department of Respiratory Medicine, The Affiliated Hospital of Putian University, No.999 Dongzhen East Road, Pu’tian, 351100 Fujian People’s Republic of China

**Keywords:** Cancer models, Lung cancer

## Abstract

Lung cancer is the most malignant form of cancer and has the highest morbidity and mortality worldwide. Due to drug resistance, the current chemotherapy for lung cancer is not effective and has poor therapeutic effects. Tripchlorolide (T4), a natural extract from the plant *Tripterygium wilfordii*, has powerful immunosuppressive and antitumour effects and may become a potential therapeutic agent for lung cancer. Therefore, this study aimed to investigate the effect of T4 on reducing chemoresistance in lung cancer cells and to explore the mechanism. 1. A549 and A549/DDP cells were separately transfected with AEG-1 overexpression and AEG-1 knockdown plasmids. A549/DDP cells were divided into the A549/DDP empty group, T4 group, and T4 + AEG-1 overexpression group. A CCK-8 assay was used to evaluate the proliferation of cells in each group. RT–qPCR and Western blotting were used to detect the expression of AEG-1 and MDR-1. Expression of AEG-1 in A549 and A549/DDP cells was positively correlated with cisplatin resistance. When the AEG-1 protein was overexpressed in A549 cells, the lethal effect of cisplatin on A549 cells was attenuated (all P < 0.05). After the AEG-1 protein was knocked down in A549/DDP cells, cisplatin was applied. The lethal effect was significantly increased compared to that in the corresponding control cells (all P < 0.05). AEG-1 protein expression gradually decreased with increasing T4 concentration in A549 and A549/DDP cells. Resistance to cisplatin was reduced after the addition of T4 to A549/DDP cells (P < 0.05), and this effect was enhanced after transfection with the AEG-1 knockdown plasmid. T4 plays an important role in increasing the sensitivity of lung cancer cells to cisplatin.

## Introduction

Lung cancer is the most malignant form of cancer and has the highest morbidity and mortality worldwide. Most patients are already in the middle and advanced stages when they are diagnosed; thus, chemotherapy has become the first therapeutic option for most patients. However, clinical data show that chemotherapy for lung cancer, especially non-small-cell lung cancer (NSCLC), is not effective or is effective only in the initial treatment period, and it is difficult to achieve the desired effect with repeated treatment. The main reason for this difficulty is the resistance of tumours to chemotherapeutic drugs^1^, especially multidrug resistance (MDR), which is a major cause of clinical treatment failure.

Drug resistance, especially multidrug resistance (MDR), is a major cause of clinical failure in cancer therapy. For most drugs, resistance develops during clinical treatment via inhibition of drug transport proteins, such as p-glycoprotein (P-gp), which mediates tumour MDR^2^. Effectively reducing the expression of the MDR1 gene can increase the sensitivity of chemotherapy patients to chemotherapeutic drugs, and this has become a hot topic in recent years.

Studies have shown that astrocyte upregulated gene 1 (AEG-1) is a key factor promoting the development of tumours^2^. Abnormal expression of the protein encoded by AEG-1 can regulate the malignant transformation of cells through various signalling pathways, promote the proliferation and antiapoptotic abilities of tumour cells, facilitate the invasion and metastasis of tumour cells, and accelerate tumour development^3^. Elevated AEG-1 expression leads to enhanced phenotypic characteristics of malignant invasion in tumour cells, including an increased proliferative capacity, invasion of surrounding tissues, migration, induction of neovascularization, and enhancement of tumour resistance^4^, while inhibition of AEG-1 by siRNA not only reduces the metastasis and invasion of human glioma cells^5^ but also reduces the expression of the MDR1 gene, thereby decreasing the resistance of tumours to chemotherapeutic drugs^6^. Therefore, the identification of strategies for effective inhibition of AEG-1, which is expected to reduce MDR1 protein expression and increase the sensitivity of tumours to chemotherapeutic drugs, is essential^2^.

Tripchlorolide (T4) has a powerful immunosuppressive effect and anti-inflammatory activity. In our previous studies, we found that T4 can attenuate the expression of MDR1, thereby increasing the sensitivity of A549/DDP cells to cisplatin^7^. T4 induces autophagy in lung cancer cells by inhibiting the PI3K/AKT/mTOR pathway and improves cisplatin sensitivity in A549/DDP cells. Therefore, in this experiment, we aimed to observe the correlation between AEG-1 expression and drug resistance in lung cancer cells, investigate the effect of T4 on the expression of AEG-1/MDR1 in lung cancer cells, and clarify the mechanism and targets of T4 related to chemotherapeutic resistance in lung cancer.

## Materials and methods

### Materials and reagents

Tripchlorolide (T4) was purchased from Amresco (Amresco, CA, USA).CCK-8 kit was purchased from Dojin-do (Dojin- do, Japan), and dimethyl sulfoxide (DMSO), protease inhibitor cocktail and TEMED were purchased from Sigma (St. Louis, MO, USA). Protein quantitative BCA kit and RIPA lysate were purchased from Beyotime.30% acrylamide was purchased from Xiamen Lu Long Company and Ammonium persulfate (AP) from Amresco Company; Pre-stained Protein Ladder and RNA reverse transcription kit were purchased from Thermo Scientific. Western Blot chemiluminescence assay kit was purchased from KPL. High glucose medium, 1640 medium, penicillin and streptomycin were purchased from Hyclone and fetal bovine serum from GEMINI.

### Cell culture

A549 and A549/DDP lung cancer cells (cisplatin-resistant lung cancer cell lines) were obtained from the Cell Line Bank, Chinese Academy of Sciences. A549 cells were cultured in DMEM-high glucose supplemented with10% fetal bovine serum (FBS) and 100 μg/mL penicillin/streptomycin in a humidified incubator under 5% CO_2_ at 37 °C. A549/DDP cells were cultured in RPMI-1640 supplemented with 10% fetal bovine serum and 100 μg/mL penicillin/streptomycin in a humidified incubator under 5% CO_2_ at 37 °C. The cell culture media were replaced with fresh media every two days.

### Drug treatments

A549 and A549/DDP lung cancer cells were seeded in 6-well plates at a density of 4 × 10^5^ cells per dish and randomly divided into the following five groups: (i) the control group, with no drug treatment; (ii–v) T4 group, exposed to T4 at a series of concentration of 25, 50, 100 and 200 nM. The cells were collected after they were treated with/without T4 for 24 h. According to the results of our previous study^8^, the IC50 of A549 and A549/DDP cells is 200 nM after 24 h; therefore, we chose 200 nM for 24 h as the reaction concentration for the subsequent experiments. The optimum T4 concentration is selected. Each experiment was performed in triplicate.

### Silencing and overexpression

Cells were seeded into 6-well plates. 24 h later, the culture medium was replaced with serum-free medium. Plasmid complementary DNA AEG-1 cDNA (pcDNA-AEG-1) was obtained by introducing AEG-1 cDNA sequence into the pcDNA3.1 expression vector. The shRNA and siRNA targeting AEG-1 and its controls were synthesized by Genepharma Company (Shanghai, China). Oligonucleotide and plasmid transfection was performed by using Lipofectamine 2000 Reagent (Invitrogen). AEG-1 siRNA:

s: 5’-GGUGAAGAUAACUCUACUGUU-3’,

as: 5’-CAGUAGAGUUAUCUUCACCUU-3’.

### CCK-8 assay

Approximately 1 × 10^5^ cells were plated into a 96-well plate and cultured for 24 h. A549 cells or A549/DDP cells were digested in logarithmic growth phase and treated with cell suspension without FBS (excluding the effect of FBS on cell proliferation). 200 L cell suspensions were taken from 5 × 10 4 /mL each and inoculated in 96-well plates. The cells were cultured for 24 h and covered with the bottom of the well.T4 was dissolved in DMSO and the experimental group, control group and blank group were set. The experimental group was divided into 6 subgroups. Serum free T4 medium containing 0, 25, 50, 100 and 200 nM was added. The cells of the control group were cultured in serum-free medium and only DMSO was added. The blank group only had medium and DMSO and no cells. After twenty-four hours, CCK8 was added to each well, and the plate was re-incubated at 37 °C for 1–4 h. The absorbance value was analyzed at 595 nm with a microplate reader. The cell viability rate was calculated according to the following formula: viability rate = A595(experimental group)/A595(control group) × 100%. Viability rate = A595/DDP(experimental group)/A595/DDP(control group) × 100%. Each experiment was performed in triplicate.

### Western blotting

The total protein extracted from cells was isolated using RIPA lysis buffer with 1 mM PMSF and kept on ice for 10 min followed by 15 min of centrifugation at 12 000 rpm and 4 °C, and the protein con-centration was measured using the BCA protein assay kit. 40 ng of protein was separated by sodium dodecyl-sulfate–polyacrylamide gel electrophoresis (SDS-PAGE) and transferred to a polyvinylidene difluoride membrane. After blocking with 5% nonfat milk for 2 h, the membranes were incubated with the primary antibody against AEG-1 (1: 1000, Abcam, USA), MDR-1 (1: 1000, Abcam, USA) and GAPDH (1: 2000, Abcam, USA), overnight at 4 °C, and followed by subsequent incubation with the respective secondary antibodies for 2 h at room temperature. After washing the blots three times with TBST, they were visualized using electrochemiluminescence plus reagent. Finally, the intensities of these blots were quantified using Image Lab 3.0 software (Bio-Rad).

### Quantitative real-time PCR

The total RNA of A549 and A549/DDP cells were extracted using the TRIzol reagent. 1000 ng of total RNA was reverse transcribed to synthesize cDNA. To quantify real-time PCR (qPCR), a total of 10 μL of the reaction volume was used, including 5 μL of 2 × SYBR Master Mix, 0.25 μL of each primer and 4.5 μL of diluted cDNA. The parameters of RT-PCR were: 10 min at 95 °C, followed by 40 cycles of 15 s at 95 °C and 1 min at 60 °C. The reaction was performed using a CFX96 Real-Time PCR System (Bio-Rad). The cycle threshold (C_t_) values were obtained and normalized to the level of GAPDH. The level of relative mRNA of each target gene was calculated using the 2^−∆∆Ct^ method. The primer sequences are as follows:

AEG-1 forward, 5'-TGTGTGTCCGTCTACAGATGTG-3',

AEG-1 reverse, 5'-TCGGCAGGAAGTGTGATTGG-3';

MDR-1 forward, 5'-TTGCCAACCATAGATGAAGG-3',

MDR-1 reverse, 5'-CACCACTGGAGCATTGACTAC-3';

GAPDH forward,5'-GTG CCA GCC TCG TCT CA TAG-3',

GAPDH reverse,5'-CTT TGT CAC AAG AGA AGG CAG-3';

Each reaction was run in triplicate. All experiments had efficiencies between 95 and 105%, and the primers displayed normal melt curves.

### Statistical analysis

The data are expressed as the mean SD (standard deviation) of triplicates. The statistical significance of the differences throughout this study was assessed by one-way ANOVA. All analyses were conducted using SPSS 13.0 software. P-values < 0.05 were considered statistically significant.

## Results

### AEG-1 protein expression in A549 and A549/DDP cells is positively correlated with cisplatin resistance

**Figure 1:**
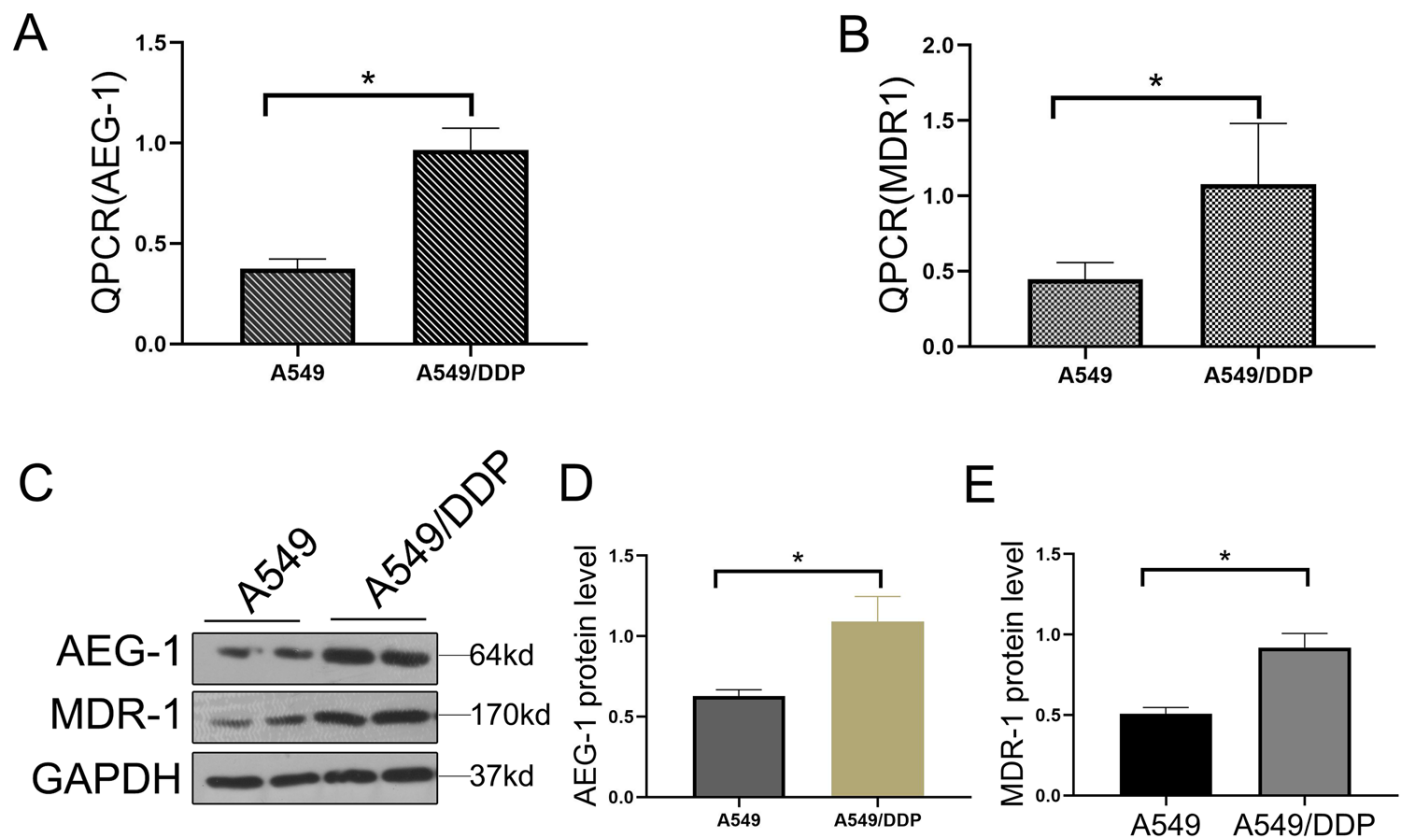
The expression of AEG-1 and MDR-1 in A549 and A549/DDP cells. (**A**) The expression of AEG-1 in mRNA level. (**B**) The expression of MDR-1 in mRNA level. (**C**) The expression of AEG-1 and MDR-1 in protein level. (**D**–**F**) Quantitative analysis of AEG-1 and MDR-1 protein expression.

**Figure 2:**
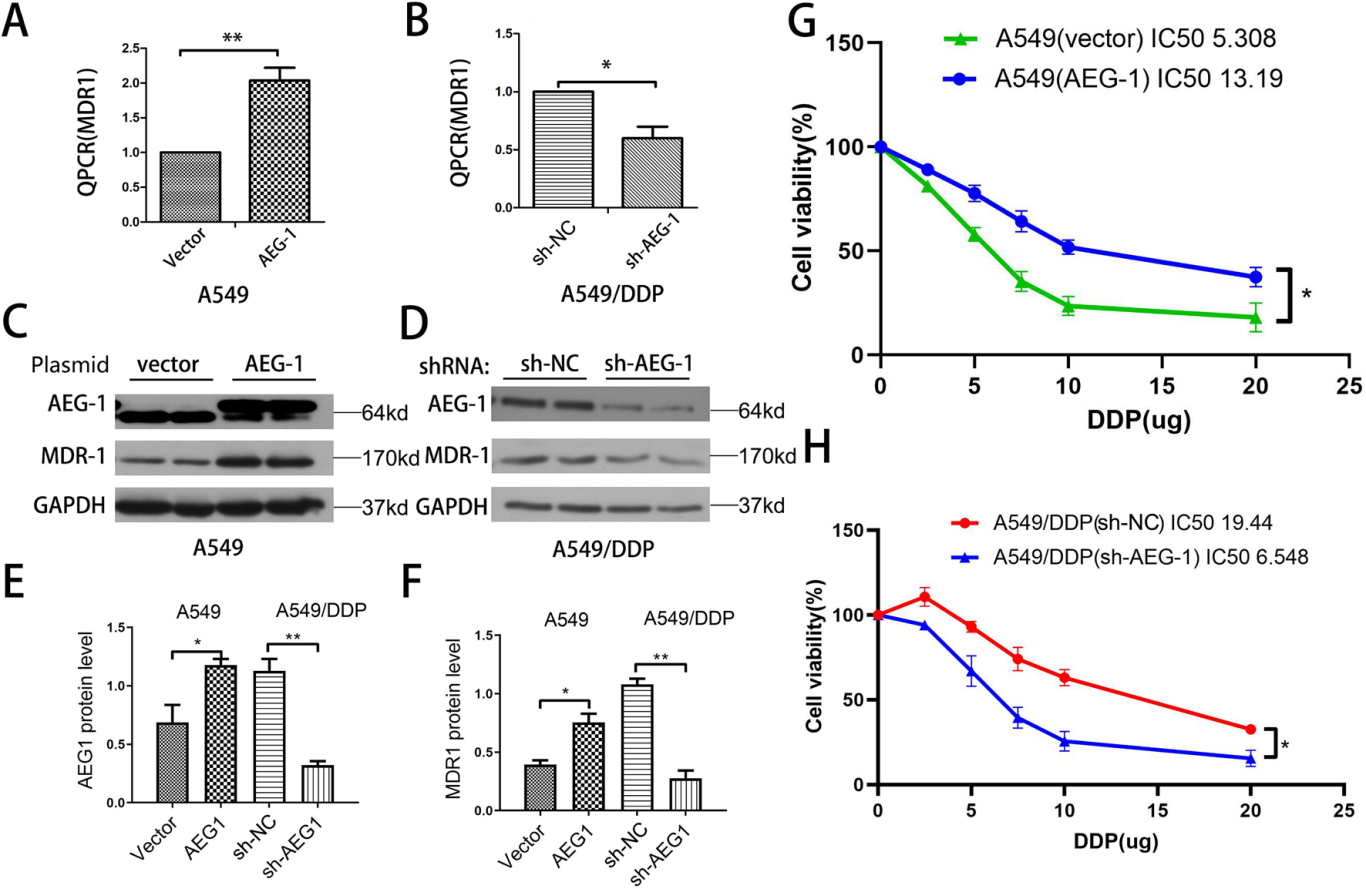
The effect of AEG-1 and MDR-1 on the killing effect of platinum in A549 and A549/DDP cells. (**A**–**D**) A549 and A549/DDP cells overexpress AEG-1 and knock down AEG-1, AEG-1 and MDR-1 respectively; (**E**,**F**) Quantitative analysis of WB results; (**G**,**H**) overexpress AEG-1 and knock down AEG-1 respectively. The killing effect of platinum on A549 and A549/DDP cells.

In this study, A549/DDP cell lines resistant to cisplatin were generated using the A549 cell line. The expression levels of AEG-1 and MDR-1 cells were detected by QPCR and WB. The results showed that AEG-1 and MDR-1 has higher expression in A549/DDP cells than those in A549 cells (Fig. 1A–E). Furthermore, The AEG-1 overexpression plasmid and sh-AEG-1 were separately transfected into A549 and A549/DDP cells. Compared with the corresponding control cells, the transfected cells exhibited significant AEG-1 overexpression or knockdown (Fig. 2C–E). The results showed that the plasmids had been transfected successfully. The mRNA and protein levels of MDR-1 increased with overexpression of AEG-1 in A549 cells. When the expression of AEG-1 was decreased in A549/DDP cells, the mRNA and protein levels of MDR-1 were also decreased (Fig. 2A,B,F). The results showed that the expression of AEG-1 and MDR-1 was closely related, which also indicated that the expression level of AEG-1 may be related to drug resistance in lung cancer cells. Therefore, we further observed the effect of AEG-1 on drug resistance in lung cancer cells through a CCK-8 assay. The results showed that when AEG-1 was overexpressed in A549 cells, the lethal effect of cisplatin on A549 cells was weakened. After AEG-1 was knocked out in A549/DDP cells, cisplatin showed a significantly enhanced lethal effect compared with that in control cells (Fig. 2G,H). Therefore, we found that the expression of AEG-1 was positively correlated with that of the drug resistance gene MDR-1 in lung cancer cells, which also showed that AEG-1 expression in lung cancer cells was positively related to cisplatin resistance.

### T4 can affect AEG-1 and MDR1 protein expression in A549 and A549/DDP cells

**Figure 3:**
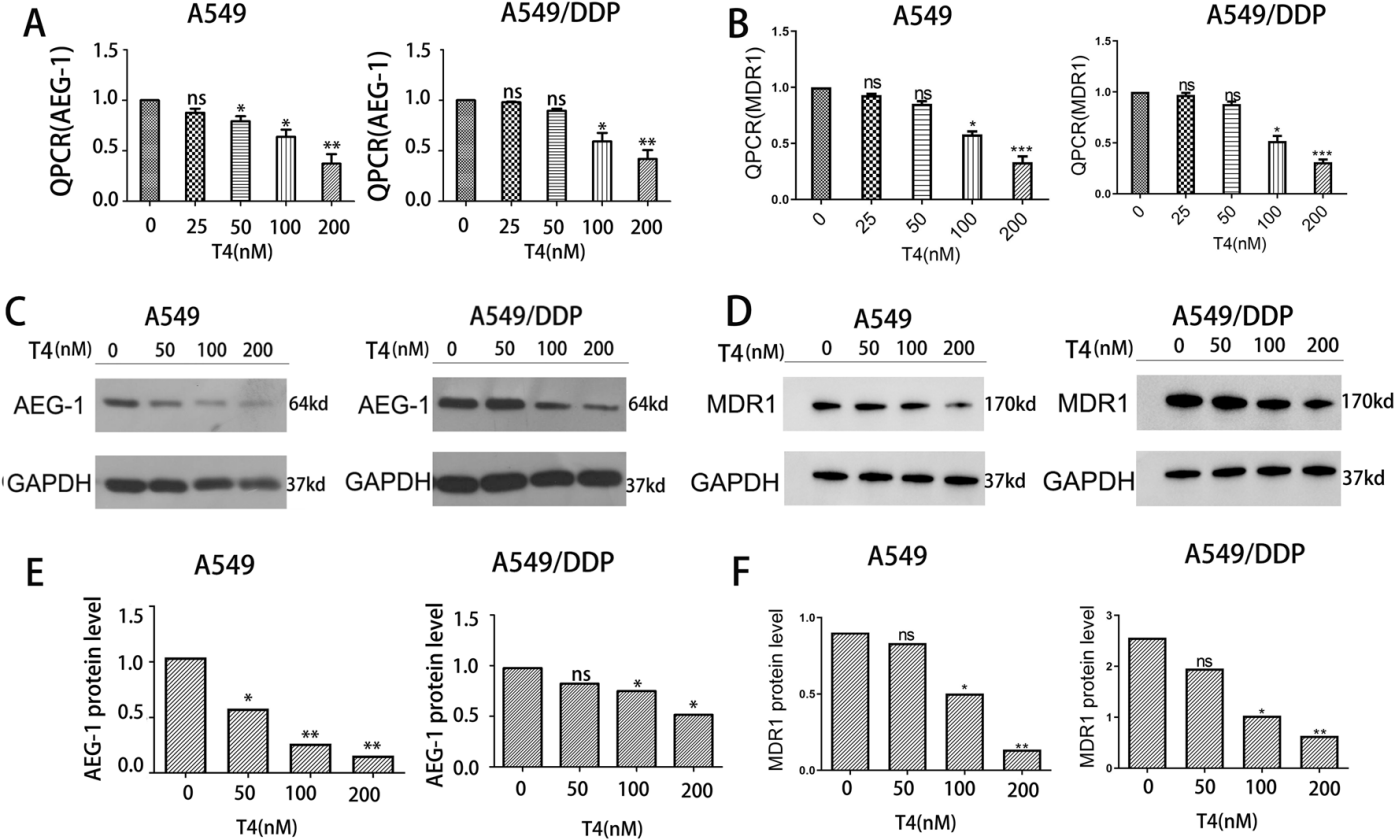
The effect of T4 on regulating AEG-1 expression. (**A**,**B**) The mRNA expression of AEG-1 and MDR-1 in T4, A549 and A549/DDP cells. (**C**,**D**) The protein expression of AEG-1 and MDR-1 in T4, A549 and A549/DDP cells. (**E**,**F**) Quantitative analysis of AEG-1 and MDR-1 protein expression.

In previous studies, it was found that T4 is related to drug resistance in lung cancer cells^7^. Therefore, in this study, the potential mechanism of T4 in lung cancer cell resistance was further explored. First, different concentrations of T4 were added to A549 and A549/DDP cells, and the expression level of AEG-1 and MDR1 was measured. As the T4 concentration increased, the expression level of AEG-1 and MDR1 in A549 and A549/DDP cells gradually decreased (Fig. 3A–F). The experimental results showed that T4 can regulate AEG-1 and MDR1 protein expression in A549 and A549/DDP cells in a dose-dependent manner. The optimal concentration of T4 was 200 nM for both groups of cells.

### T4 can affect drug resistance in lung cancer cells by affecting the expression of AEG-1

**Figure 4:**
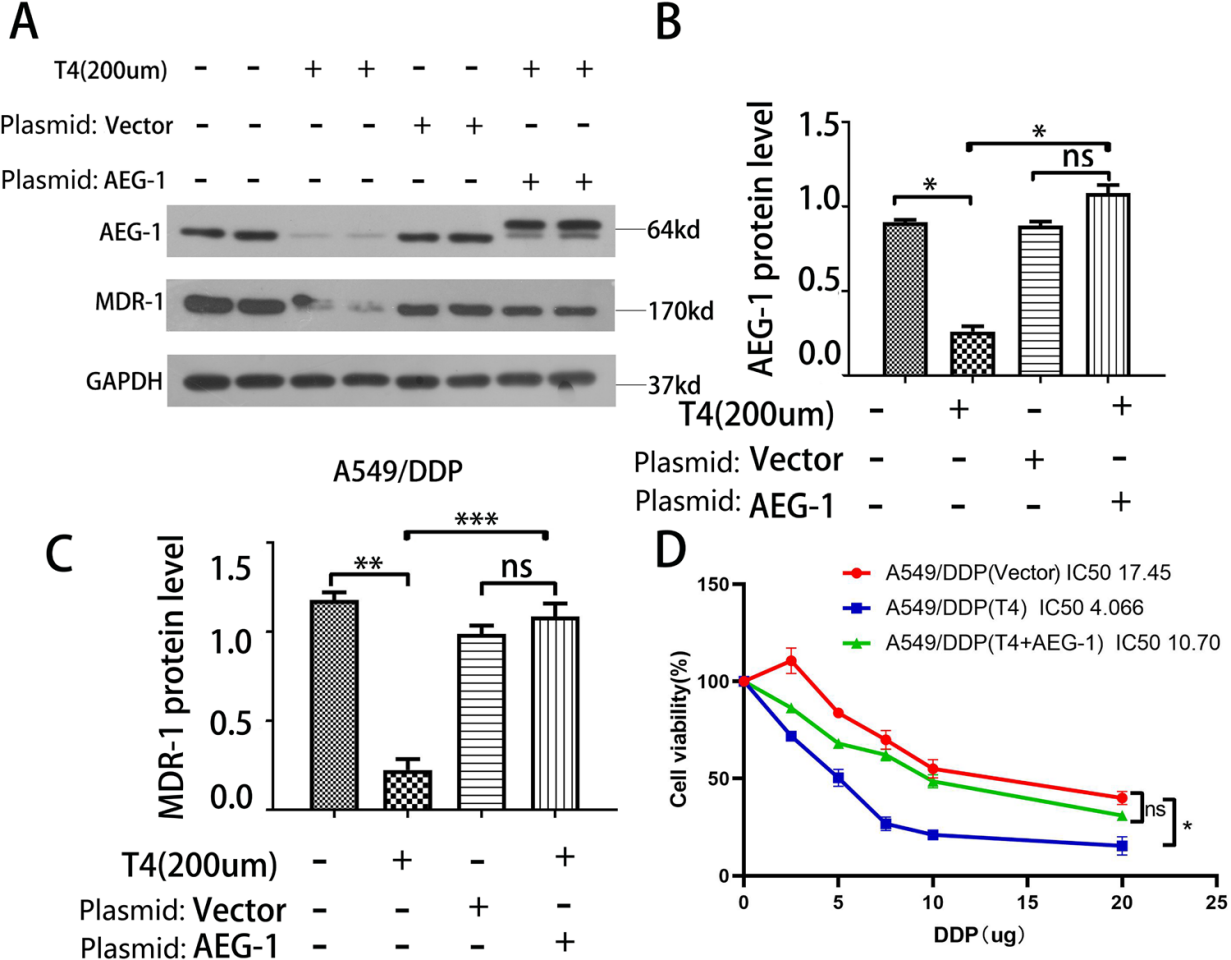
The effect of T4 on the killing effect of cisplatin. (**A**–**C**) AEG-1 and MDR-1 protein expression in A549/DDP cells; (**D**) The killing effect of cisplatin on A549/DDP cells.

After treatment of A549/DDP cells and AEG-1-overexpressing A549/DDP cells with 200 nM T4, AEG-1 protein expression was significantly reduced in T4-treated A549/DDP cells compared with that in control cells, and the expression of MDR-1 was also reduced accordingly. After both AEG-1 overexpression and T4 treatment, the expression level of MDR-1 increased significantly (Fig. 4A–C), indicating that T4 can regulate MDR-1 expression by affecting AEG-1 expression. The CCK-8 assay results showed that T4 reduced the resistance of A549/DDP cells to cisplatin. After AEG-1 was overexpressed via plasmid transfection, the effect of T4 on cisplatin resistance was weakened (Fig. 4D). These experiments showed that T4 can affect the resistance of lung cancer cells to cisplatin by affecting the expression of the AEG-1 protein.

## Discussion

AEG-1 was first reported in 2002 as a neuropathology-associated gene induced in human foetal astrocytes following human immunodeficiency virus-1 (HIV-1) infection or treatment with recombinant HIV-1 envelope glycoprotein (gp120), and it has emerged as a potentially crucial mediator of malignancy in cancers and a key node in a complex network of oncogenic signalling pathways^9,10^. One of the important hallmarks of aggressive cancers is chemoresistance. Studies have suggested that AEG-1 contributes to resistance to a broad spectrum of chemotherapeutics, including 5‑fluorouracil, doxorubicin, paclitaxel, cisplatin and 4-hydroxycyclophosphamide^9,11–13^. Cisplatin is a powerful anticancer drug. Currently, cisplatin is widely used in the treatment of various human cancers. It has significant clinical effects in various solid tumours, such as those of bladder cancer, head and neck cancer, ovarian cancer and testicular cancer^14^. Due to its strong antitumour activity, cisplatin is also the key chemotherapeutic drug for the clinical treatment of non-small-cell lung cancer. However, drug resistance often leads to the failure of chemotherapy in lung cancer. AEG-1 has been reported to be related to the resistance of tumours to chemotherapeutic drugs, with an increasing number of studies published on this matter in recent years. AEG-1 increases multidrug resistance gene 1 (MDR1) protein expression, which facilitates the association between MDR1 mRNA and polysomes, leading to increased translation, inhibition of ubiquitination and consequent proteasome-mediated degradation of the MDR1 protein^11^. Studies have shown that elevated AEG-1 expression can increase the resistance of tumours to chemotherapeutic drugs. For example, AEG-1 can increase the expression of the MDR1 gene in cancers such as liver cancer and breast cancer. AEG-1 can increase MDR1 mRNA translocation to polynucleosomes to promote translation of the MDR1 protein^11^. Lung cancer is a malignancy that is extremely resistant to chemotherapeutic drugs. Therefore, it is important to inhibit the resistance of lung cancer cells to chemotherapeutic drugs. In this study, AEG-1 expression was positively correlated with cisplatin resistance in lung cancer cells. Therefore, regulation of AEG-1 expression constitutes a new direction for reducing chemoresistance in lung cancer.

In recent years, traditional Chinese medicine treatment has been widely used in clinical practice. Medicinal plants are therapeutic resources rich in biological compounds that provide raw materials for nearly 75% of prescription drugs worldwide^15^. Among these biological compounds, the natural extract of the plant *Tripterygium wilfordii* has a strong immunosuppressive effect and anti-inflammatory activity; it has been widely used in the treatment of rheumatic diseases, kidney diseases and other autoimmune-related diseases in China. However, the severe toxicity and side effects of the crude extract of Tripterygium limit its clinical application^16^. According to previous studies, triptolide inhibits the proliferation of cancer cells in vitro and reduces the growth and metastases of tumours in vivo. However, triptolide has a potent influence on the gastrointestinal tract, skin and mucosa, reproductive system, bone marrow haematopoietic system and kidneys, and it has a significant impact on the female reproductive system. Tripchlorolide (T4) is an inhibitory monomer extracted from Tripterygium or produced by hydroxyl acylation and chlorination of the precursor compound triptolide. After structural modification, not only is the toxicity of T4 greatly reduced, its pharmacological activity is greatly improved. T4 has strong anti-inflammatory, antitumour, immunosuppressive and antifertility activities. Its toxicity is significantly lower than that of triptolide, and its chemotherapeutic index is higher than that of triptolide. In our previous study, we found that T4 can increase the sensitivity of A549/DDP cells to cisplatin by decreasing the expression of MDR-1. This study suggested that T4 can inhibit the expression of the AEG-1 gene and reduce the expression of MDR-1 via this inhibition of AEG-1 expression, thus increasing the sensitivity of lung cancer cells to cisplatin. Combined with our previous study^7^, our findings here indicate that T4 can induce autophagy by inhibiting the PI3K/AKT/mTOR pathway, thus leading to lung cancer cell death. In addition, in many studies related to autophagy and apoptosis in tumour cells, AEG-1 has been reported to be closely related to the PI3K/AKT/mTOR pathway^17^. Therefore, the specific molecular mechanism by which T4 regulates AEG-1 protein expression in lung cancer cells will be explored further in the future.

## Conclusions

In summary, our current research indicates that triptolide is important for increasing the sensitivity of lung cancer cells to cisplatin. AEG-1 may be a key protein involved in this effect and may have an important impact on the survival rate after chemotherapy in patients with lung cancer in the future. AEG-1 will be an ideal target for the development of the next generation of effective cancer therapeutics.

## Supplementary Information

Supplementary Information.

## Data Availability

All data generated or analysed during this study are included in this published article.
