# Supplementary material for: T4 reduces cisplatin resistance by inhibiting AEG-1 gene expression in lung cancer cells
Source: Sci Rep. 2022 Jul 6;12:11462. doi: 10.1038/s41598-022-15643-3 (PMC9259636; doi:10.1038/s41598-022-15643-3)

**Original WB image**

**Figure 1 C**

First and second lines from the left were protein samples from A549 cells.

Third and fourth lines from the left were protein samples from A549/DDP cells.

AEG-1


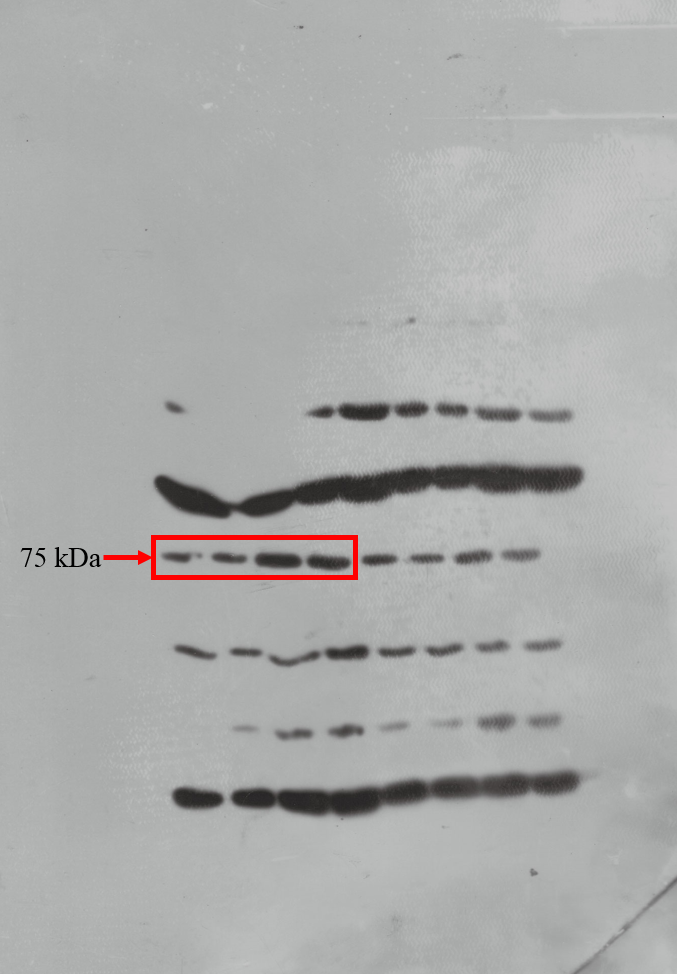


MDR-1


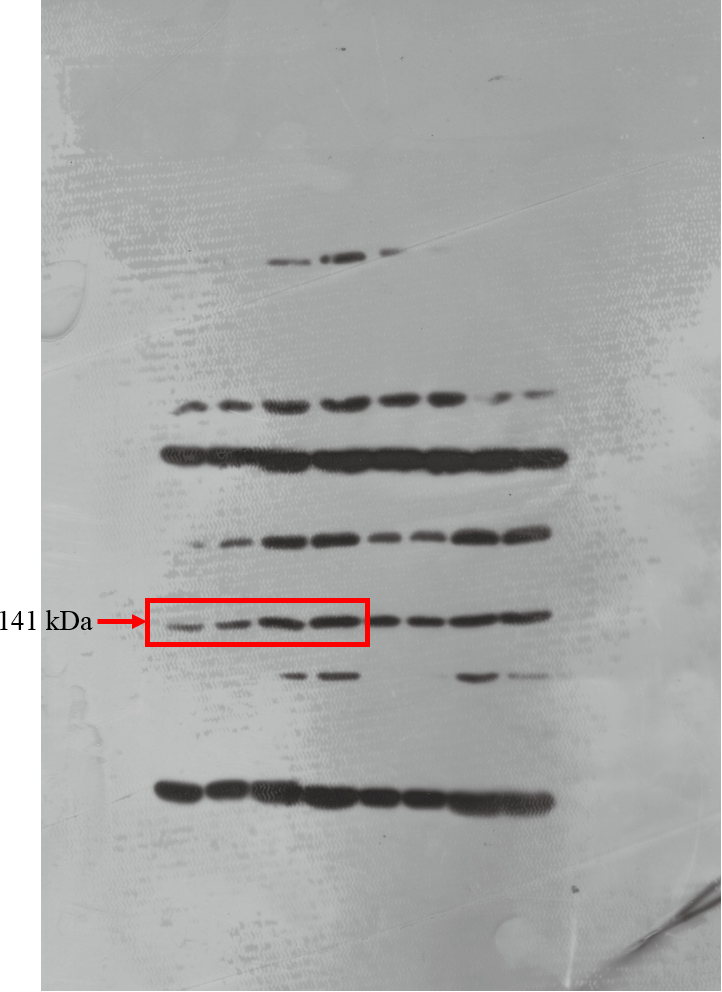


GAPDH


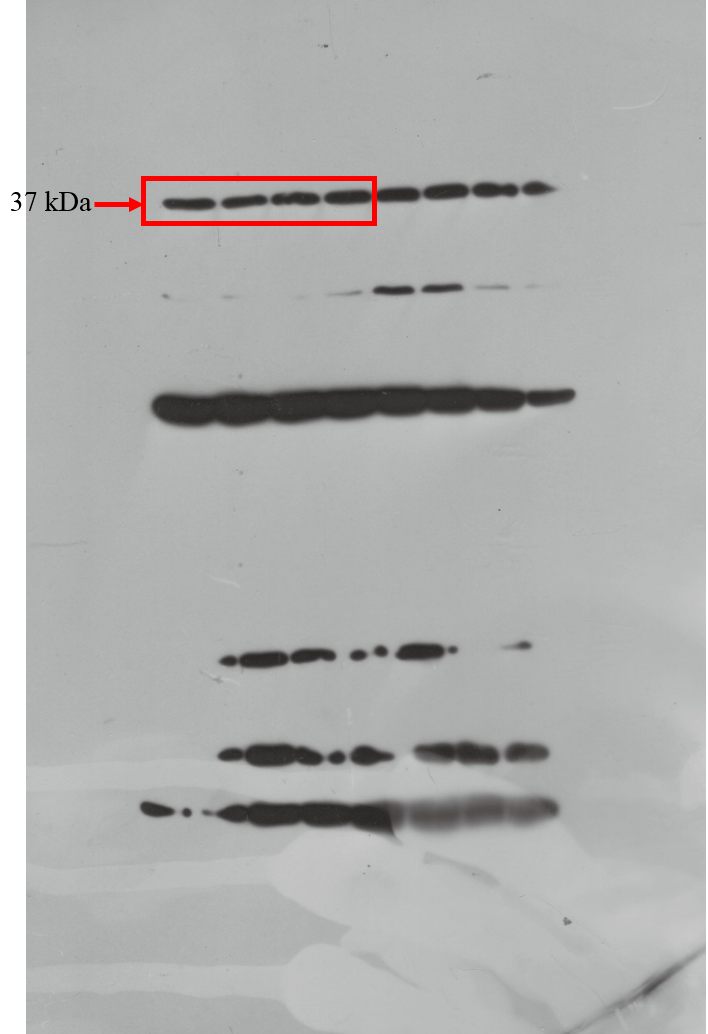


**Figure 2 C**

First and second lines from the left were protein samples from vector groups.

Third and fourth lines from the left were protein samples from AEG-1 groups

AEG-1


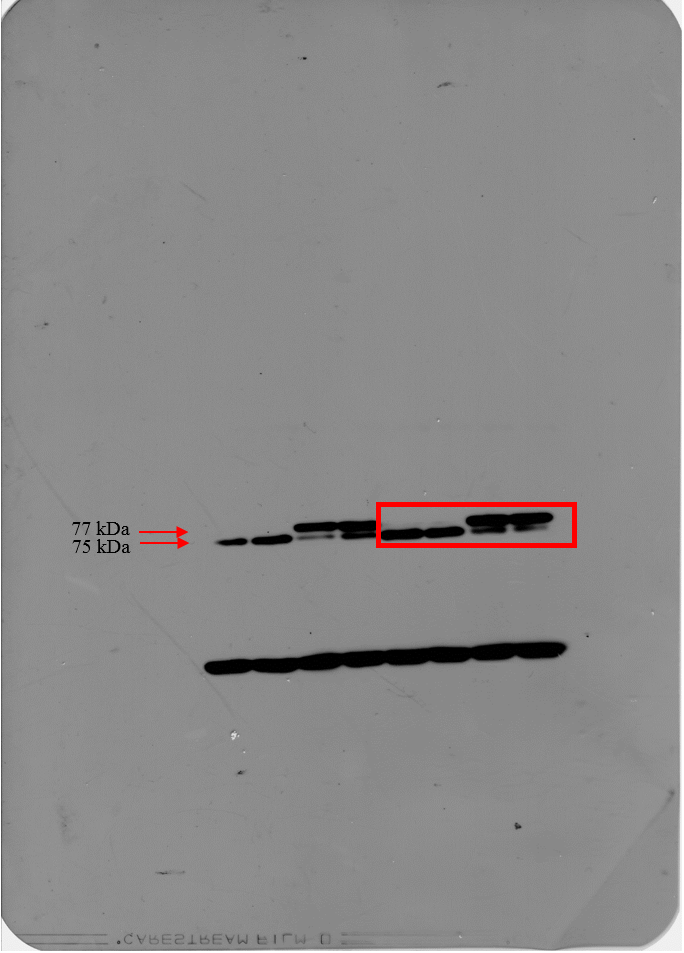


MDR-1&GAPDH

The upper line was MDR-1 and the below was GAPDH.


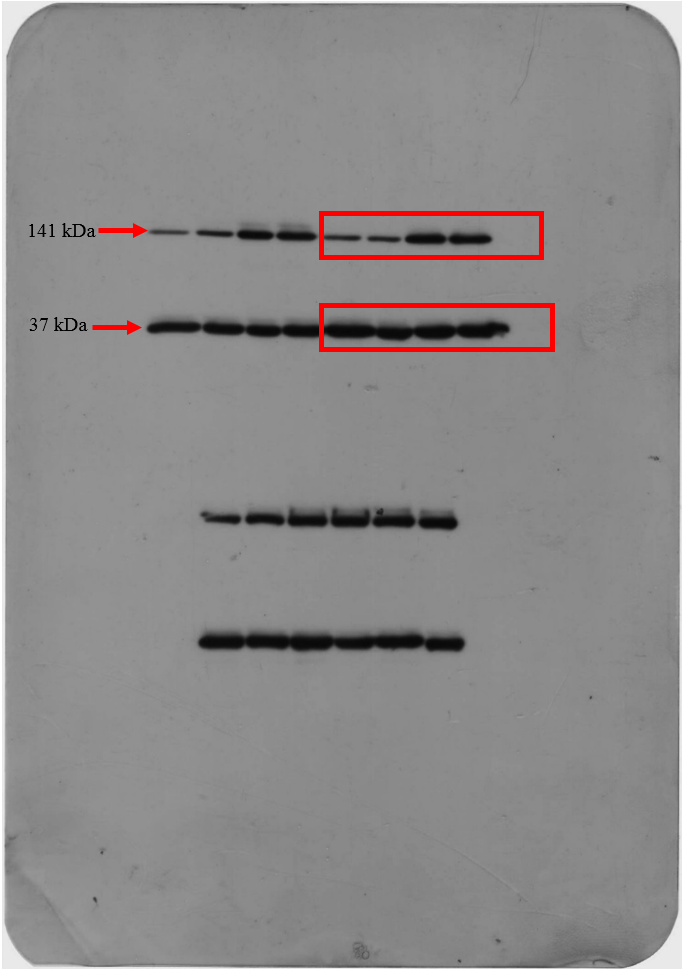


**Figure 2 D**

The proteins were AEG-1, GAPDH and MDR-1 from top to bottom. Fifth and sixth lines from the left were protein samples from sh-vector groups. Seventh and eighth lines from the left were protein samples from sh-AEG-1 groups


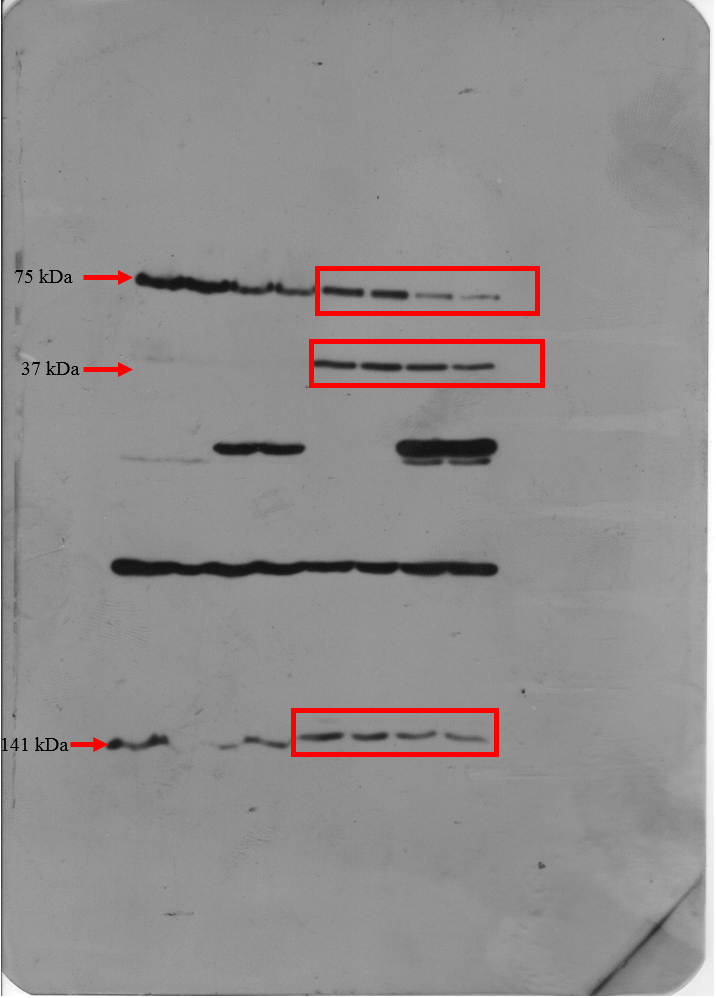


**Figure 3 C**

AEG-1&GAPDH

Fifth to eighth lines from the left were protein samples from A549 cells with different T4 concentration. The upper was AEG-1 and the below was GAPDH.


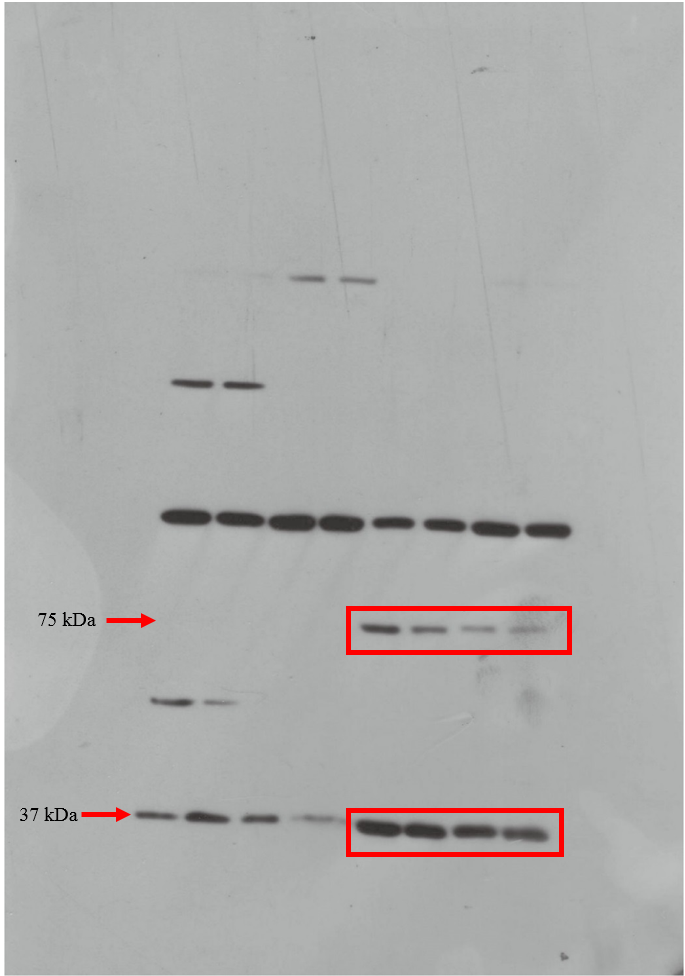


**Figure 3 D**

AEG-1

Fifth to eighth lines from the left were protein samples from A549/DDP cells with different T4 concentration.


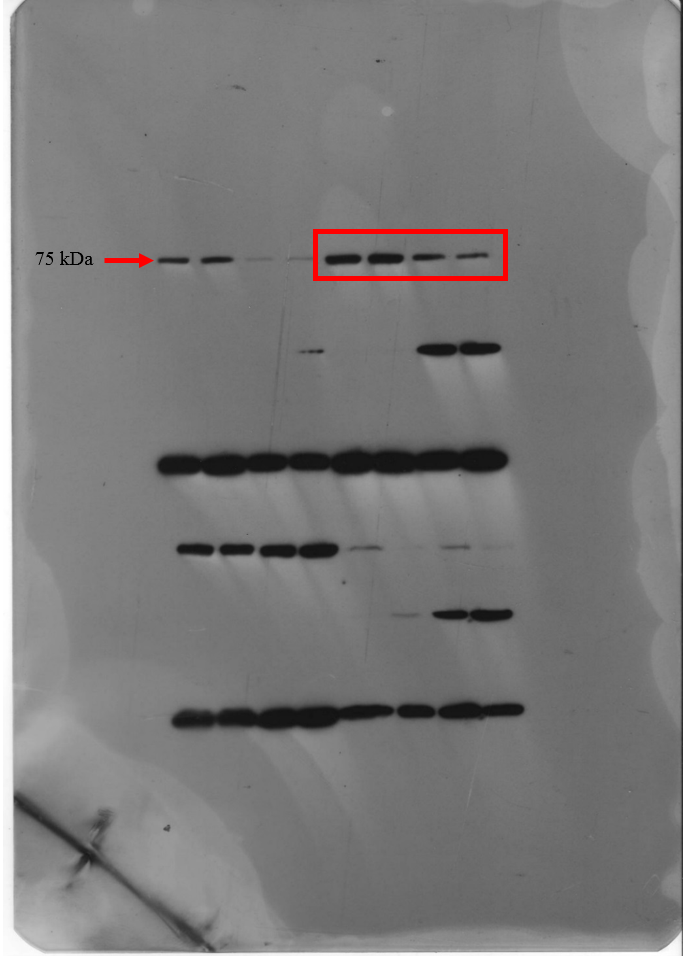


GAPDH

Fifth to eighth lines from the left were protein samples from A549/DDP cells with different T4 concentration.


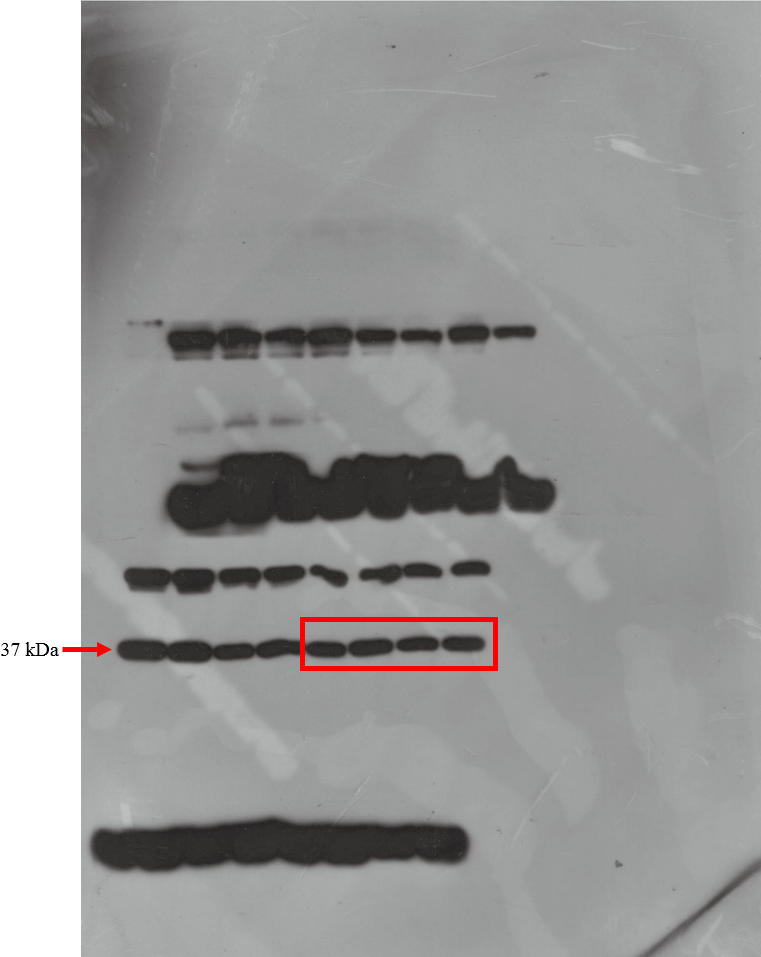


**Figure 4 A**

AEG-1


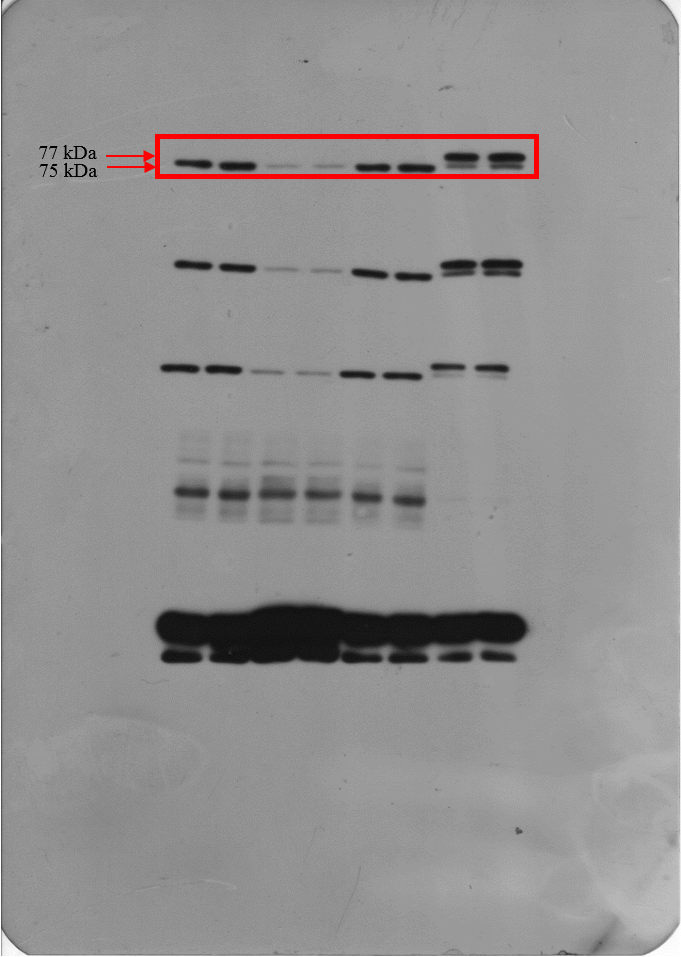


MDR1


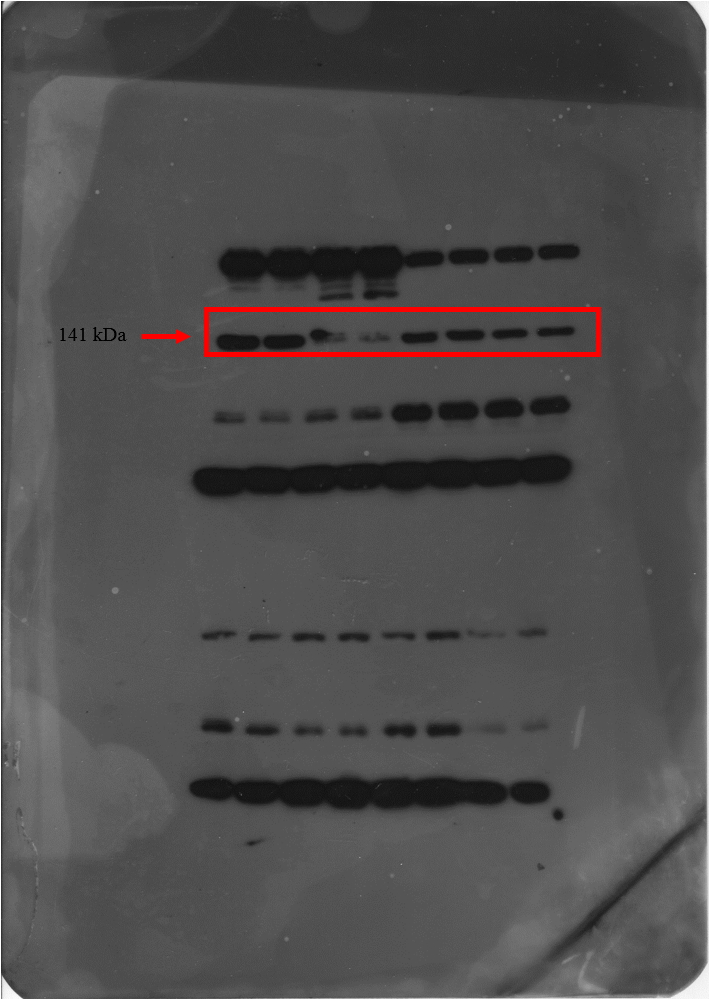


GAPDH


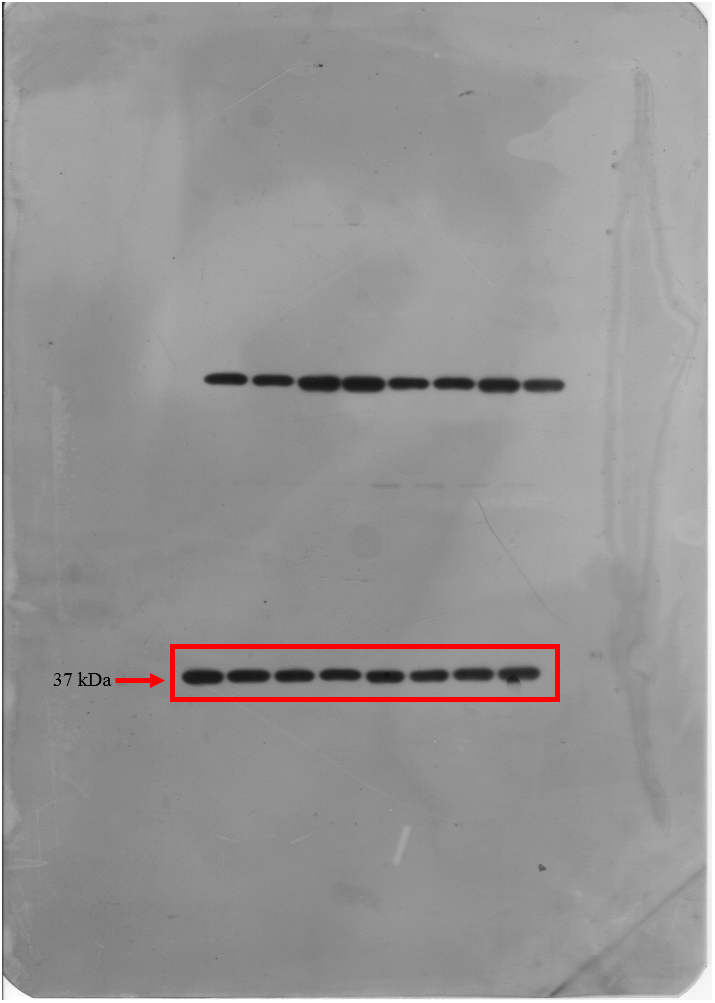

Supplement: Supplementary file 1 — Supplementary Information. [file 41598_2022_15643_MOESM1_ESM.docx]
